# Effects of steric encumbrance of iridium(iii) complex core on performance of solution-processed organic light emitting diodes†

**DOI:** 10.1039/d0ra04652c

**Published:** 2020-07-23

**Authors:** Armands Ruduss, Valdis Kokars, Natalija Tetervenoka, Aivars Vembris, Kaspars Traskovskis

**Affiliations:** Faculty of Materials Science and Applied Chemistry, Riga Technical University P. Valdena Str. 3 LV-1048 Riga Latvia kaspars.traskovskis@rtu.lv; Institute of Solid State Physics, University of Latvia Kengaraga Str. 8 LV-1063 Riga Latvia

## Abstract

Iridium(iii) complexes are the most frequently applied commercialized green and red emitters for organic light emitting diode (OLED) displays. Throughout years a significant research effort has been devoted to modify these compounds, in order to make them suitable for cost-effective solution-processing techniques, such as inkjet printing. To achieve this, the inherent tendency of the complex molecules to form poorly emissive aggregates needs to be suppressed. In many cases this has been achieved by an encapsulation of the iridium(iii) complex core with dendritic structures, composed of either passive or charge-transporting fragments. In order to validate this approach, we acquired three structural analogues of the conventional green emitter Ir(ppy)_3_, which possess gradually increasing sterical encumberment at the complex surface. Corresponding OLEDs were examined, with three distinctively different active emissive layer compositions in terms of charge transportation characteristics. The results show that in the all scenarios the unmodified Ir(ppy)_3_ outperforms the compounds with attached bulky groups. The in-device performance of the emitter is directly related to its charge trapping ability, which is being compromised in the presence of dendritic auxiliary substituents.

## Abstract 2

Attachment of bulky groups to the surface of irdium(iii) complex core obstructs its charge trapping ability and reduces OLED performance.

## Graphical abstract

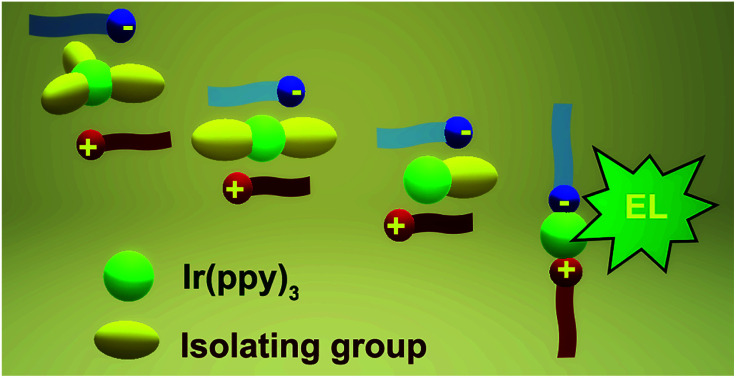

## Introduction

Organic light emitting diodes (OLED) are currently considered as the most suitable technological solution for efficient, thin, fast-refresh-rate and high image quality displays.^1^ Due to the unique properties, this technology is used in a variety of emerging innovative products, such as transparent^2^ and flexible screens.^3^ Regardless of the recent success considering the practical implementation, a transfer from the currently adopted vacuum-deposition to the much less complex solution-based manufacturing process would greatly promote the usage extent and economic viability of OLEDs.^4,5^

The widespread adoption of the wet processing mainly relies on a development of suitable light emitting molecules. Phosphorescent cyclometalated iridium(iii) complexes are the most studied and applied OLED emitters due to their efficient triplet harvesting ability, color tuning possibilities and chemical stability.^6^Ir(ppy)_3_ (tris(2-phenylpyridine)iridium)^7^ is, perhaps, the most scrutinized compound among this material class. This green emitter has been used as a model compound for numerous studies due to near to 100% photoluminescence quantum yield (*Φ*_PL_) and excellent chemical and thermal stability. When being vacuum-deposited in solid films together with a charge transporting host materials, the sparsely dispersed Ir(ppy)_3_ molecules retain *Φ*_PL_ value near unity.^8^ If the concentration of the complex is increased, the relatively large dipole moment of Ir(ppy)_3_ (ref. 9) is inducing a solid-state aggregation.^10,11^ The subsequent formation of poorly emissive excimers together with increased probability of triplet–triplet annihilation (TTA) leads to a diminished OLED performance and considerable efficiency roll-off at large current densities.^10,12^ In the solution-processed films the aggregation of Ir(ppy)_3_ is even more pronounced, causing large-scale phase separation between emitter and host materials.^13,14^ Again, this is governed by the attracting force, as the aggregation process between individual Ir(ppy)_3_ molecules is proven to take place even in solutions.^15^

As a consequence, wet-processed OLEDs based on Ir(ppy)_3_ show notably reduced performance characteristics in comparison to vacuum deposited analogues. Considerable effort has been made to chemically modify this emitter in order to overcome its limitations. In most cases this has been attempted by following an obvious path, where bulky groups with purely isolating or charge transporting functionality are being introduced to Ir(ppy)_3_ core to provide a physical barrier that suppress the aggregate formation and helps to sustain large distance between individual emitter molecules. Accordingly, Ir(ppy)_3_ has been attached to side chains of hole transporting polymers.^16,17^ Because of a well-defined structure, easier purification and reproducible synthesis of the resulting materials, the encapsulation with dendronic structures is the most frequently investigated molecular design. Structures composed of passive groups such as polyphenylenes^18–20^ and carboranes^21^ or hole transporting polycarbazoles^22^ have been extensively investigated. Despite a large number of the screened materials, none of the approaches has demonstrated clear benefits in terms of OLED performance, as it notably falls behind the vacuum-deposited or sometimes even solution-processed devices composed of unmodified Ir(ppy)_3_.^23^ No clear design rules have yet been established that could guide the development of a new-generation of efficient solution-processable OLED emitters. In particular, the set of requirements regarding the right balance of sterical encumberment and charge transportation characteristics of the materials needs to be established.

We investigate Ir(ppy)_3_ and its three structural analogues with a gradually increasing number of attached electronically passive groups. A device integration of the compounds was performed by exploring three different active emissive layer (EML) composition scenarios: with hole-transporting, balanced or predominantly electron-transporting characteristics. The results give a deeper insight about excitation mechanism in Ir(ppy)_3_ based OLEDs. Based on these results the preferable structural prerequisites can be proposed towards efficient solution-processable iridium(iii) complexes.

## Experimental section

### General procedures

Starting materials and solvents were acquired from commercial suppliers and were used without additional purification. NMR spectra were obtained on a Bruker Avance 300 MHz or Bruker Avance Neo 500 MHz spectrometer using solvent residue as an internal reference. The elemental analysis was carried out using a Costech Instruments ECS 4010 CHNS–O Elemental Combustion System. Differential scanning calorimetry (DSC) thermograms were acquired using a Mettler Toledo DSC-1/200 W apparatus at a scanning rate of 10 °C min^−1^ while keeping the samples under N_2_ atmosphere. Optical measurements were carried in tetrahydrofuran (THF) solutions with typical material concentrations of 1–4 × 10^−5^ mol L^−1^. Solutions for *Φ*_PL_ and emission decay measurements were prepared in glovebox under Ar atmosphere using previously degassed solvents. Films for optical measurements were prepared using spin-coating technique with a Laurell WS-400B-6NPP/LITE spin coater on quartz slides, using solutions with material concentration of 30 mg mL^−1^. After the preparation all films were dried in an oven at 100 °C for 2 h. The UV-vis spectra were recorded with a PerkinElmer Lambda 35 spectrometer. Emission spectra, *Φ*_PL_ and emission lifetimes were determined using QuantaMaster 40 steady state spectrofluorometer (Photon Technology International, Inc.) equipped with 6 inch integrating sphere (LabSphere). The excitation wavelength in the all cases was 410 nm. The molecular ionization potential (IP) and photoconductivity measurements (*E*_th_) were carried out on a self-made experimental system, using a procedure described in our previous work.^24^ Density functional theory (DFT) calculations for geometry optimization and excited state energies were performed using ORCA^25^ program package (build 4.0.1.2). For auxiliary tasks Avogadro program was used.^26^ DFT and TDDFT calculations employed non-local functional B3LYP with def2-TZVP^27^ basis set. [SD(60,MWB)]^28^ effective core potentials (ECP) were used for iridium atom. To reduce the computational time, the structures were simplified by substituting 3,3,3-triphenylmethyl propionic acid with acetic acid fragment.

## Results and discussion

### Synthesis

**Scheme 1:**
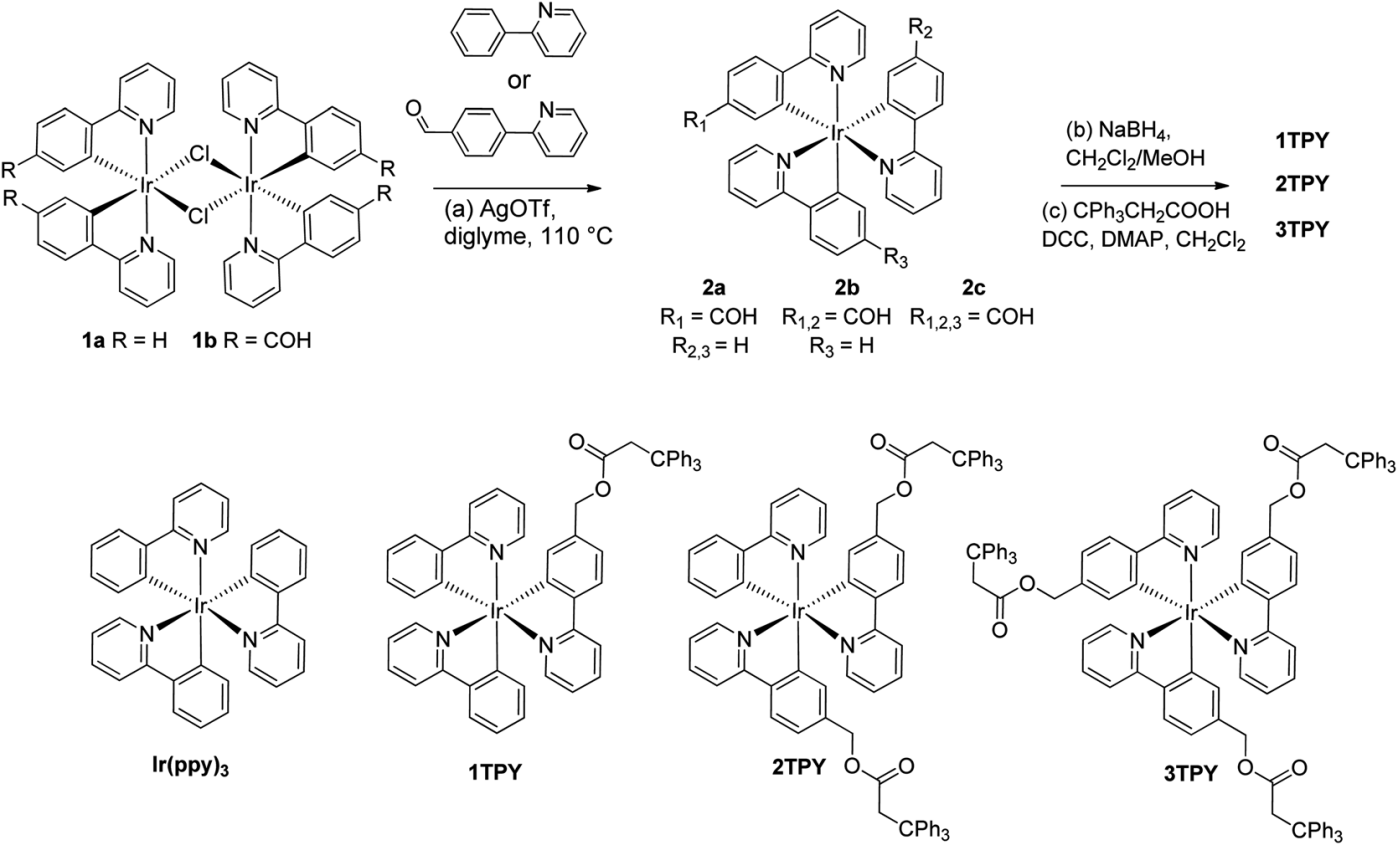
Synthesis of the investigated structural analogues of Ir(ppy)_3_.

For the purpose of the study three novel structural analogues of Ir(ppy)_3_ were acquired according to Scheme 1. The compounds feature the emissive Ir(ppy)_3_ core and one (1TPY), two (2TPY) or three (3TPY) attached bulky triphenylmethane groups (TR). In order to minimalize the impact of the substituents on the photophysical properties of the emitter, the linkage between TR and the complex core is realized through an aliphatic bridge. Such approach has been previously applied to acquire solution-processable emitters with a solely purpose to provide a sterical barrier between complex molecules.^29^ The synthesis was performed using μ-chloro-bridged iridium(iii) complex dimers 1a^30^ and 1b^31^ as the starting materials. The introduction of the third cyclometalating ligand was accomplished by utilizing the procedure by Colombo *et al.*,^32^ which strictly yields tris-cyclometalated complexes with a *fac*-structural configuration. Among the performed synthetic series, the rate and the yield of the cyclometalation reaction increases with the number of electron accepting benzaldehyde fragments in the resulting complex. The target compounds were acquired after the reduction of the aldehyde groups and subsequent esterification of the resulting alcohols with 3,3,3-triphenylpropionic acid.

NMR spectra and the elemental composition of the synthesized materials are consistent with the proposed structures. ^13^C spectra of 3TPY indicate a chemical equivalence of 2-phenylpyridine (ppy) ligands and ancillary groups, underlining *C*_3_ symmetry of the complex core. The symmetry is being broken in the case of 1TPY and 2TPY, resulting in an observation of three chemically inequivalent sets of ppy signals.

### Characterization of emitters

**Fig. 1:**
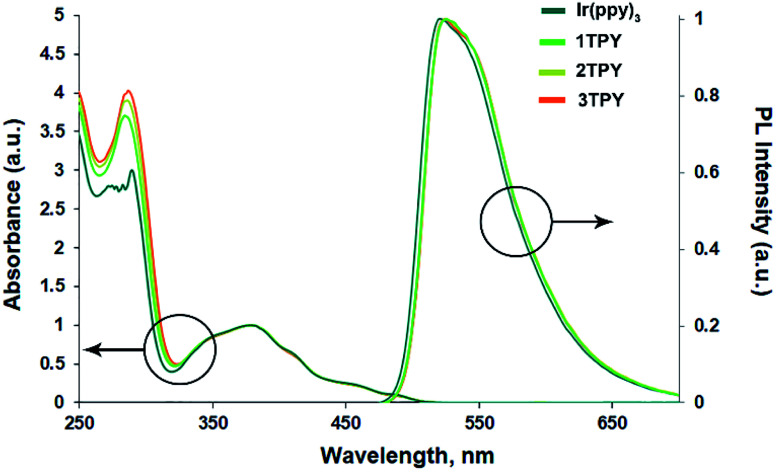
UV-vis absorption and emission spectra of the compounds in THF.

The UV-vis absorption and emission spectra of Ir(ppy)_3_ and its three modified analogues are given in Fig. 1. No major deviations can be observed between the compounds, suggesting that the attachment of TR groups has not significantly altered the electronic configuration of Ir(ppy)_3_ core. In the region below 300 nm UV-vis absorption bands show increased absorbance with the number of attached TR groups, indicating an overlap between π–π* transitions of ppy ligands and TR phenyl rings. In the region between 300–500 nm, which features spin allowed metal-to-ligand charge transfer (^1^MLCT) and the corresponding triplet transitions (^3^MLCT),^33^ the bands overlap with only minor deviations.

In deoxygenated THF solution the emission bands of the compounds assume an identical broad featureless shape. A bathochromic shift of the emission by 3 nm can be observed for TR-functionalized compounds in respect to the emission maximum of Ir(ppy)_3_ (at 522 nm). *Φ*_PL_ values of the compounds (Table 1) vary in the range of 0.94–0.90. A slight drop in the emission efficiency can be observed for TR-functionalized compounds, that can be associated with an increased molecular complexity that promotes the probability of non-radiative decay pathways. Emission decay times show very little variation between the compounds and lay in the interval 1.7–1.8 μs. Radiative (*k*_r_) and non-radiative (*k*_nr_) decay rates are also close, although Ir(ppy)_3_ shows a slightly higher *k*_r_ and lower *k*_nr_ values.

**Table 1:** Photophysical properties of the investigated emitters

Compound	λ_max em._, nm	*Φ*_PL_ a	*τ*, μs	*k*_r_ b, ×10^5^ s^−1^	*k*_nr_ c, ×10^5^ s^−1^	IP, eV	EA, eV	HOMO^d^, eV	LUMO^d^, eV	Δ*E*_S0–S1_^d^, eV	Δ*E*_S0–T1_^d^, eV
Ir(ppy)_3_	522	0.94/—	1.7	5.5	0.35	5.15	2.85	−5.06	−1.47	2.85	2.66
1TPY	525	0.91/0.01	1.8	5.1	0.50	5.17	2.95	−5.15	−1.57	2.83	2.63
2TPY	525	0.90/0.03	1.7	5.3	0.58	5.21	2.95	−5.24	−1.66	2.84	2.64
3TPY	525	0.93/0.08	1.8	5.2	0.38	5.24	2.92	−5.32	−1.74	2.85	2.65

a Measured in deoxygenated THF/amorphous films.

b Radiative decay rate *k*_r_ = *Φ*_PL_/*τ*.

c Nonradiative decay rate *k*_nr_ = (1 − *Φ*_PL_)/*τ*.

d Calculated values.

**Fig. 2:**
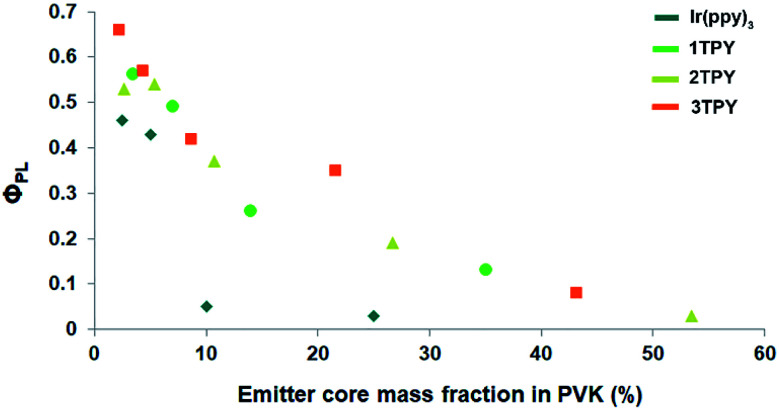
*Φ*_PL_ values of spin-coated films composed of PVK host and Ir(ppy)_3_-based emitters at different emitter mass fractions. For TR-functionalized compounds only the mass of the emitter core is counted towards the emitter mass content.

The functionalization of Ir(ppy)_3_ with TR groups causes distinctive changes in a solid-state morphology of the corresponding compounds, as the unmodified complex is crystalline but its derivatives are amorphous. DSC analysis of the synthesized compounds reveal only one distinctive phase transition signal – glass transition temperature (*T*_g_) (Fig. S1†). The measured *T*_g_ values of the compounds 1TPY, 2TPY and 3TPY are, accordingly, 158, 142 and 131 °C, indicating that the presence of additional TR groups causes a drop in softening temperature of the materials. This can be attributed to an increased overall conformational freedom of the molecular fragments,^34^ as TR fragments are bound to the complex core through a flexible linker. Despite the lack of crystallinity, the amorphous spin-coated films of the corresponding compounds show poor *Φ*_PL_, with the best value of 0.08 in the case of 3TPY, indicating a strong emission quenching. While 3TPY has a large number of attached TR groups that should partly prevent close contacts, the facial configuration of the complex core determines that only a half of its octahedral surface area is being sterically shielded. In order to deeper investigate the aggregation tendency between the compounds, emitter concentration impact on *Φ*_PL_ in guest–host systems with poly(9-vinylcarbazole) (PVK) was determined (Fig. 2). The results show that the presence of shielding groups has a minor impact on *Φ*_PL_ when the concentration of the emitters is below 10 wt%. Still, a little positive effect of the TR substituents can be seen in comparison to the unmodified complex. Above 10 wt% Ir(ppy)_3_ shows a steep drop in *Φ*_PL_ in comparison to the modified compounds. This can be associated with a large-scale phase separation and formation of poorly emissive crystalline inclusions. TR-functionalized compounds, on the other hand, show a gradual decrease in *Φ*_PL_ due to the lack of crystal formation ability. Still, the ongoing aggregation is apparent. Taking into consideration the partly-shielded structure of the complex molecules, the formation of predominantly dimeric aggregated species is assumed instead of large-scale mass separation in the case of Ir(ppy)_3_.

With an increasing mass concentration, the compounds generally show a broadening and redshift of the emission bands (Fig. S2, Table S1†). Notable transformations in the emission bands can be detected even at 5 wt% emitter content, indicating already ongoing aggregation. At 50 wt% the emission in the all cases originate mainly from excimers, with the emission maxima located in the range of 544–551 nm.^12^ At concentrations exceeding 20 wt% 3TPY shows contrasting emission band transformations, with a less distinctive full width at half maximum (FWHM) increase. This can be attributed to the partly shielded molecular surface of the compound that limits the number of possible spatial arrangements of aggregated complex molecules. The structure of 3TPY also determines that only pyridine ring of TR-ppy ligands can form direct close contacts, in such way reducing the extent of energy level shifts in the formed excimers.

**Fig. 3:**
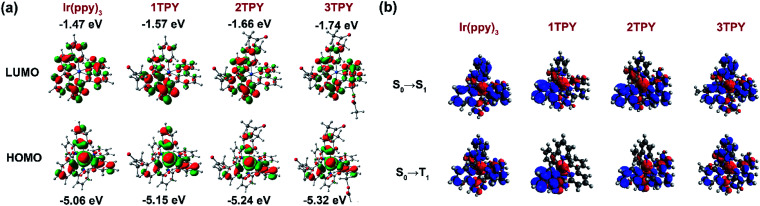
(a) Calculated configuration and energies of HOMO and LUMO orbitals. (b) Calculated electron density difference maps for the lowest singlet and triplet excitations. Electron transfer proceeds from the red to blue regions.

In order to interpret the slight variations in photophysical properties between the compounds, density functional theory (DFT) calculations were performed. The distribution of the frontier molecular orbitals and their energies are outlined in Fig. 3a. The configuration of the highest occupied molecular orbital (HOMO) are similar in all the cases, with electron density being mainly localized on iridium d orbitals and the electron rich phenyl rings of the three ppy ligands. Regarding the lowest unoccupied molecular orbital (LUMO), it is evident that for TR-functionalized compounds LUMO resides on the TR-modified ppy ligand. As a result, LUMO in 1TPY is localized on a single but in 2TPY on two ligands, breaking the symmetry of the molecule. In 3TPY, similarly to Ir(ppy)_3_, LUMO is evenly distributed across all three ppy ligands. The localization of LUMO on the functionalized ppy ligands can be attributed to a weak negative inductive effect of the present benzyl ester substituent. Such substitution pattern is shown to stabilize LUMO level in ppy-type cyclometalating ligands.^35^ TDDFT calculations were used to predict the lowest singlet (Δ*E*_S0–S1_) and triplet (Δ*E*_S0–T1_) transition energies (Table 1). In the all cases the obtained values show little variations, with the difference not exceeding 0.02 eV for singlets and 0.03 eV for triplets. A slight lowering in triplet level is predicted for all TR-functionalized compounds, this being consistent with the experimentally observed redshift of the corresponding phosphorescence bands. Electron density difference maps for the lowest singlet excitation predict that this process is mainly associated with an electron density shift between HOMO and LUMO orbitals (Fig. 3b). As a result, the excitation is localized on two ligands for 2TPY and one ligand for 1TPY, in comparison to the involvement of all ligands in *C*_3_-symmetric Ir(ppy)_3_ and 3TPY. Among the TR-functionalized compounds a correlation between the number of TR-ppy ligands and Δ*E*_S0–S1_ and Δ*E*_S0–T1_ can be observed, where Δ*E* for the transitions gradually increases with the number of the modified ligands. This increase in transition energy can be attributed to the growing degeneracy of the excited states.^36^

Previous experimental and theoretical studies of the emissive properties of Ir(ppy)_3_ have shown that in experimental setting the *C*_3_ symmetry of the complex is being reduced and the phosphorescence proceeds from *C*_1_-symmetric excited triplet state, which is localized only on one of the cyclometalating ligands.^33,36–38^ The main cause for this process is Jahn–Teller distortion, which lifts the energetically unfavorable degeneracy of the long-lived *C*_3_-symmetric excited state through a transformation of the molecular geometry.^36–38^ An interaction with surrounding solvent or host matrix molecules is also considered as a possible origin for the loss of the symmetry.^33^ The aforementioned processes are also expected to take place in the case of the investigated derivatives of Ir(ppy)_3_. The manifestation of this effect explains the closely similar emissive properties of 1TPY, 2TPY and 3TPY despite the variations in their structural composition. The phosphorescence in all these compounds is expected to originate from an excited state that lies on a single TR-functionalized ppy ligand. This localization of the *T*_1_ excitation is caused by the more stabilized LUMO level of TR-ppy (for 1TPY) in a combination with a lift in the degeneracy of the *T*_1_ state (for 2TPY and 3TPY).

The electronic levels in thin films of the investigated emitters were determined by photoemission yield spectroscopy, to find ionization potential (IP), and photoconductivity measurements, to determine photoconductivity threshold value (*E*_th_). Electron affinity (EA) was then calculated as the difference between IP and *E*_th_ (Table 1). The determined IP and EA values correlate well with the calculated HOMO and LUMO energies, as IP and EA assume slightly deeper energies with an increased number of the attached TR groups.

All the previous measurements indicate that the studied compounds possess highly similar emissive properties both in solution and films and can be used as appropriate model compounds to investigate the influence of sterical encumberment on the OLED performance.

### Electroluminescence properties

**Fig. 4:**
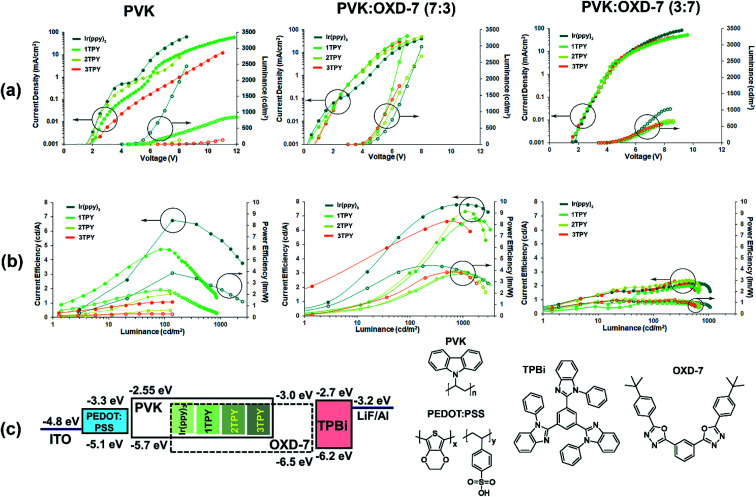
OLED characteristics for devices with three different composition EMLs. (a) Voltage–current density (filled circle) and luminance (empty circle) plots. (b) Luminance–current efficiency (filled circle) and power efficiency plots. (c) Energy diagram and the used materials featuring experimentally obtained IP and EA values.

In order to evaluate the electroluminescence characteristics of the compounds, three-layer OLEDs composed of hole transporting PEDOT:PSS, emissive layer and electron transporting TPBi were examined. Spin-coated EMLs were composed of the Ir(ppy)_3_ or one of the three TR-functionalized emitters that were dispersed in a charge transporting host material. The Ir(ppy)_3_ complex core content in the all cases were set at 7 wt%, attributing the mass of TR groups to the host. Three configurations of EML were studied, in which the host material was predominantly hole-transporting PVK, a balanced mixture of PVK and electron-transporting OXD-7 (70 : 30 wt%) or a mostly electron-transporting blend of the same two materials (30 : 70 wt%). The device characteristics expressed in voltage–current density–luminance and luminance–current efficiency (*η*_c_)–power efficiency (*η*_p_) plots together with energy diagram are outlined in Fig. 4. Turn on voltage, maximal efficiency, luminance and roll-off parameters are given in Table 2.

**Table 2:** Characteristics of OLEDs with PVK, PVK : OXD-7 (70 : 30 wt%) or PVK : OXD-7 (30 : 70 wt%) EML host materials

Compound	*V*_on_ a, V	*η*_c_ b, cd A^−1^	*η*_p_ c, lm W^−1^	*L*_max_ d, cd m^−2^	Roll-off^e^, %	CIE^f^ (*x*, *y*)
**PVK**						
Ir(ppy)_3_	4.5	6.8	3.9	2446	44	0.32, 0.62
1TPY	4.5	4.8	2.4	844	71	0.35, 0.58
2TPY	6	1.6	0.6	127	—	0.32, 0.62
3TPY	7	1.0	0.3	136	—	0.34, 0.59
**PVK** **:** **OXD-7 (7** **:** **3)**						
Ir(ppy)_3_	3.5	7.8	4.4	2983	6	0.31, 0.63
1TPY	4	6.9	3.7	3308	10	0.31, 0.63
2TPY	4	7.3	3.9	2692	27	0.32, 0.62
3TPY	3.5	6.6	3.8	1794	10	0.31, 0.63
**PVK** **:** **OXD-7 (3** **:** **7)**						
Ir(ppy)_3_	3.5	2.2	1.1	1048	27	0.30, 0.63
1TPY	3.5	2.0	1.0	638	23	0.31, 0.62
2TPY	3.5	2.4	1.3	680	28	0.32, 0.62
3TPY	4	2.3	1.3	560	25	0.32, 0.62

a Turn-on voltage.

b Current efficiency.

c Power efficiency.

d Maximal luminance.

e Current efficiency drop between the maximal value and the value at the highest luminance.

f Measured at maximal brightness.

For the devices with PVK host a clear correlation between the chemical composition of the emitter and performance can be observed, where an increasing sterical encumberment of the complex surface leads to a poorer electroluminescence. For Ir(ppy)_3_ the maximal brightness of the device reaches 2500 cd m^−2^, while for 2TPY and 3TPY this parameter barely surpasses a 100 cd m^−2^ mark. The maximal attained efficiency (*η*_c_ and *η*_p_) also decreases in accordance with the amount the attached TR groups. For a predominantly hole-transporting EML, such as PVK, it is expected that the emission originates in a close proximity to the interface of EML and electron transport layer, where holes can recombine with locally trapped electrons. In the particular case, the electrons are expected to reside mainly on the emitter molecules, due to a closer energetic level alignment with the electron transporting layer and about 0.4 eV deeper electron affinity level in respect to PVK. Further electron transfer between the closely packed complex molecules is not expected due to the poor charge mobilities of Ir(ppy)_3_ complex core.^39,40^ A notably increased device charge density can be observed in the case of Ir(ppy)_3_. This indicates that the complex molecule is directly aiding the electron transfer into EML. For TR-functionalized compounds such process is obstructed due to the present isolating groups, leading to lower current densities and barely detectable electroluminescence, as the charge recombination zone is moved to the electron transport layer. The electroluminescence in devices bearing Ir(ppy)_3_ and 1TPY emitters is accompanied with a steep and severe efficiency roll-off. This is another consequence of a narrow recombination zone. Due to the high localization of the emitting molecules the electroluminescence is being quenched by TTA almost immediately after the emission onset.

OLEDs with EMLs composed of 70 : 30 wt% mixture of PVK and electron transporting material OXD-7 exhibit somewhat similar performance level across the all investigated emitters. At the same time a considerable efficiency improvement can be seen in a comparison with PVK-only devices. Due to a less restricted charge injection, the luminance onset values are significantly lowered, varying in the range of 3.5–4.5 V. Among the emitters, Ir(ppy)_3_ still performs the best in terms of *η*_c_ and *η*_p_, with 3TPY being the least efficient compound. This indicates that sterical shielding is obstructing electroluminescence process even in scenario, where molecules are surrounded by charge carriers of the both types. In a stark contrast to the devices with pure PVK host, the lowest current density is observed in the case of Ir(ppy)_3_ emitter. This can be attributed to a more pronounced charge trapping on Ir(ppy)_3_ molecules due to a direct exposure of the molecular surface to surrounding host molecules. Such process is aiding the radiative recombination and reduces leaked current, leading to increased current efficiency of the device. The much lower extent of efficiency roll-off, in a comparison to the previously characterized devices, indicates a much broader charge recombination zone.

Finally, OLEDs with a predominantly electron transporting mixture of host materials, PVK : OXD-7 (30 : 70 wt%), were examined. Among the investigated series, these devices exhibit the worst overall efficiency and luminance parameters. Regarding the emitter chemical composition, the least variations can be observed between the structures. The only notable difference is a slightly increased maximal attainable brightness in the case of Ir(ppy)_3_. The analysis of the previously discussed OLEDs strongly suggests that charge trapping by emitter molecules is a crucial process towards attainment of efficient devices. This has been demonstrated previously for closely related iridium complexes, where the excitation of an emitter by charge trapping is clearly dominating over other possible excitation pathway – Förster energy transfer from excited host molecules.^41^ Taking this into account, the lowered performance for the OXD-7 enriched OLEDs can be attributed to the excessive electron transport across EML. This results in a charge recombination zone being moved either at the interface with or inside hole injection layer. In an analogy with PVK-only devices, one would expect that the reduced hole transportation ability of OXD-7 saturated EML would promote a hole trapping process on the emitter molecules. Contrary, the minimal difference in the performance between the emitter structures suggest that an initial trapping of holes play a little role in the excitation process of the studied emitters, as such process would be clearly affected by sterical effects. This reduced tendency to host holes can be explained by about 0.5 eV deeper ionization potential of PVK in respect to the emitter molecules that effectively contains holes on the host material.

## Conclusions

A series of three structural analogues of Ir(ppy)_3_ was acquired, where compounds possess a gradually increasing sterical encumberment at the surface of the emitting complex core. The photophysical properties and solid-state behavior of the synthesized compounds are almost identical to those of the unmodified complex, providing an appropriate basis for OLED performance benchmarking. OLEDs with solution-processed EMLs, with three different configurations of charge transporting host materials, were investigated. The acquired results indicate that the electroluminescence process in the devices is mainly driven by an initial charge trapping on the emitter molecules. As a result, Ir(ppy)_3_ outperforms the emitters with attached bulky groups due to an unrestricted exposure of the complex surface to the neighboring charge transporting molecules.

The obtained results suggest that material design strategy towards efficient iridium(iii) based solution-processable OLED emitters should avoid the use of passive isolating groups, as the resulting structures perform even worse the than unmodified complex molecules. At the same time, it is clear that the strong tendency for aggregation within the compound class sets a requirement for a complete sterical encapsulation. The use of charge-transporting dendritic peripheral groups is an extensively investigated direction towards achieving this, but several shortcomings can be identified regarding this approach. First, the highly unordered structure of such dendritic fragments makes charge transportation within them unlikely, as close packing and regular crystal-like arrangement of the molecules is needed to realize an efficient charge transportation process.^42^ As a result, in-device performance of such materials is not expected to be much different from the emitters with electronically passive peripheral groups. Second, such hypothetical charge transporting groups should provide pathways towards charge trapping on the emitter core by an appropriate energy level tuning. Unrestricted charge transport would result in a recombination zone shift towards charge transporting layers, similarly to the process observed in our examined OXD-7 saturated OLEDs.

Based on our findings, the use of isolating peripheral groups with a charge trapping functionality can be proposed as a promising design strategy. Such OLEDs should be composed of EMLs with carefully arranged electronical levels, where the sterical groups at the surface of the emitter would act as an intermediate charge trap, located energetically between the levels of the charge transporting host and the emissive core.

## Conflicts of interest

There are no conflicts to declare.

## Supplementary Material

RA-010-D0RA04652C-s001
