# Supplementary material for: Effects of steric encumbrance of iridium(iii) complex core on performance of solution-processed organic light emitting diodes
Source: RSC Adv. 2020 Jul 23;10(46):27552–9. doi: 10.1039/d0ra04652c (PMC9055592; doi:10.1039/d0ra04652c)
Supplement: RA-010-D0RA04652C-s001 [file RA-010-D0RA04652C-s001.pdf]

Electronic Supplementary Material (ESI) for RSC Advances.
This journal is © The Royal Society of Chemistry 2020

Supporting information for

Effects of steric encumbrance of iridium(III) complex core on performance of solution-processed organic light emitting diodes

Armands Ruduss,[a] Valdis Kokars,[a] Natalija Tetervenoka,[b] Aivars Vembris,[b] Kaspars Traskovskis[a]*

[a] Faculty of Materials Science and Applied Chemistry, Riga Technical University, P. Valdena Str. 3, LV-1048, Riga, Latvia

[b] Institute of Solid State Physics, University of Latvia, Kengaraga Str. 8, LV-1063, Riga, Latvia

*Corresponding author: e-mail: kaspars.traskovskis@rtu.lv

Contents

Experimental procedures 3

Synthesis 3

NMR spectra of the synthesized compounds 6

OLED preparation and characterization 9

Figure S1 11

Figure S2. 12

Table S1. 13

References 14

Experimental procedures

Synthesis

General synthesis method of aldehyde group containing homoleptic iridium(III) complexes. A mixture of the corresponding μ-hloro-bridged iridium(III) complex dimer (0.4 mmol), cyclometalating ligand (4.0 mmol) and silver triflate (0.8 mmol) in diglyme (4 mL) was placed in a round bottom flask. The reaction vessel was then deoxygenated by multiple vacuum-argon purge cycles and the reaction mixture was heated at 110 °C for 4 h under an argon atmosphere. The product was precipitated by an addition of ethanol. The obtained solid was collected by filtration and purified by column chromatography using CH_2Cl_2/EtOAc (10:1, v/v) as an eluent.

Iridium(III) complex 2a. The compound was obtained from μ-hloro-bridged iridium(III) complex dimer (1a)[1] and 4-(2-pyridyl)benzaldehyde as an orange powder. Yield: 39%. ^{1}H NMR (300 MHz, $CDCl_3$) δ (ppm): 6.60 (d, J = 6.0 Hz, 1H), 6.65 – 6.96 (m, 7H), 7.14 – 7.21 (m, 2H), 7.32 – 7.48 (m, 3H), 7.48 – 7.65 (m, 6H), 7.68 (d, J = 8.1 Hz, 1H), 7.82 (dd, J = 8.1. Hz, J = 2.3 Hz, 2H), 7.90 (d, J = 8.1 Hz, 1H), 9.62 (s, 1H).

Iridium(III) complex 2b. The compound was obtained from μ-hloro-bridged iridium(III) complex dimer (1b)[2] and 2-phenylpyridine as an orange-red powder. Yield: 58%. ^{1}H NMR (300 MHz, $CDCl_3$) δ (ppm): 6.68 (d, J = 7.6 Hz, 1H), 6.73 – 6.89 (m, 3H), 6.89 – 6.97 (t, J = 5.6 Hz, 2H), 7.13 (d, J = 1.4 Hz, 1H), 7.21 (d, J = 1.4 Hz, 1H), 7.34 – 7.45 (m, 3H), 7.49 (d, J = 5.0 Hz, 1H), 7.54 (d, J = 6.3 Hz, 2H), 7.56 – 7.64 (m, 3H), 7.65 – 7.73 (m, 2H), 7.83 (d, J = 8.1 Hz, 1H), 7.91 (d, J = 7.8 Hz, 1H), 9.59 (s, 1H), 9.63 (s, 1H).

Iridium(III) complex 2c. The compound was obtained from μ-hloro-bridged iridium(III) complex dimer (1b) and 4-(2-pyridyl)benzaldehyde as a red powder. Yield: 64%. ^{1}H NMR

(300 MHz, $CDCl_3$) δ (ppm): 6.95 (td, J = 6.8 Hz, J = 1.1 Hz, 3H), 7.12 (d, 7.12, J = 1.5 Hz, 3H), 7.42 (dd, J = 8.1 Hz, J = 1.5 Hz, 3H), 7.50 (d, J = 5.2 Hz, 3H), 7.66 (td, J = 7.4 Hz, J = 1.4 Hz, 3H), 7.73 (d, J = 8.1 Hz, 3H), 7.96 (d, J = 8.1 Hz, 3H), 9.61 (s, 3H).

General synthesis method of triphenylpropionic ancillary group containing homoleptic iridium(III) complexes. The corresponding tris-cyclometalated iridium(III) complex (0.5 mmol) was dissolved in a mixture of CH_2Cl_2 /MeOH (5 mL, 1:1, v/v) after what $NaBH_4$ (1.5 equiv.) was added to the reaction vessel. The mixture immediately changed a color from orange to yellow. After 10 min water was added in order to precipitate the product. The yellow precipitate was filtered off, washed with water and dried to yield the corresponding benzyl alcohol-functionalized iridium(III) complex in a quantitative yield. The obtained product (0.4 mmol) was dissolved in CH_2Cl_2 (10 mL) and 3,3,3-triphenylpropionic acid (2 equiv.), 4-dimethylaminopyridine (0.01 equiv.), N,N'-dicyclohexylcarbodiimide (2.1 equiv.) were sequentially added to the solution. The mixture was stirred for 24h and then filtered. Ethanol (50 mL) was added to the filtrate and CH_2Cl_2 was fractionally removed under reduced pressure. The resulting yellows solid was collected by filtration and purified by column chromatography using CH_2Cl_2 as the eluent.

1TPY. The product was obtained as a yellow powder. Yield: 87% over 2 steps. ^{1}H NMR (300 MHz, $CDCl_3$) δ (ppm): 3.59 (s, 2H), 4.51 (s, 2H), 6.49 (d, J = 8.1 Hz, 1H), 6.56 (s, 1H), 6.63 – 6.83 (m, 9H), 7.04 – 7.19 (m, 15H), 7.35 – 7.62 (m, 9H), 7.71 – 7.81 (m, 3H). ^{13}C NMR (126 MHz, $CDCl_3$) δ (ppm): 170.69, 166.30, 160.80, 147.07, 146.67, 143.38, 136.66, 136.07, 135.77, 129.25, 127.76, 126.13, 123.85, 121.93, 119.33, 118.84, 66.51, 55.72, 46.40. Anal. calcd for $C_{55}H_{42}IrN_3O_2$: C, 68.16; H, 4.37; N, 4.34. Found: C, 67.97; H, 4.63; N, 4.40.

2TPY. The product was obtained as a yellow powder. Yield: 85% over 2 steps. ^{1}H NMR (300 MHz, $CDCl_3$) δ (ppm): 3.57 (s, 4H), 4.38 – 4.58 (m, 4H), 6.43 (d, J = 8.1 Hz, 1H), 6.51 (d, J = 8.1 Hz, 1H), 6.56 (s, 1H), 6.60 (s, 1H), 6.65 – 6.81 (m, 6H), 6.98 – 7.21 (m, 30H), 7.35 – 7.54 (m, 9H), 7.69 – 7.79 (m, 3H). ^{13}C NMR (126 MHz, $CDCl_3$) δ (ppm): 170.80, 170.71, 166.71, 166.34, 166.30, 161.19, 161.03, 160.70, 147.08, 147.05, 147.04, 146.74, 146.66, 143.57, 143.46, 143.33, 137.16, 136.66, 136.61, 136.02, 136.00, 135.54, 129.96, 129.30, 129.25, 127.76, 127.74, 126.15, 126.13, 123.93, 123.78, 121.96, 121.91, 121.90, 119.99, 119.30, 119.09, 118.81, 66.62, 66.38, 55.77, 55.72, 46.46, 46.44. Anal. calcd for $C_{77}H_{60}IrN_3O_4$: C, 72.05; H, 4.71; N, 3.27. Found: C, 72.34; H, 4.93; N, 3.45.

3TPY. The product was obtained as a yellow powder. Yield: 62% over 2 steps. ^{1}H NMR (300 MHz, $CDCl_3$) δ (ppm): 3.52 (s, 6H), 4.40 (d, J_{AB} = 12 Hz, 3H), 4.49 (d, J_{AB} = 12 Hz, 3H), 6.42 (dd, J = 8.0 Hz, J = 1.5 Hz, 3H), 6.52 (d, J = 1.1 Hz, 3H), 6.76 (td, J = 6.7 Hz, J = 1.1 Hz, 3H), 7.03 – 7.19 (m, 45H), 7.34 – 7.44 (m, 6H), 7.49 (td, J = 7.7 Hz, J = 1.2 Hz, 3H), 7.74 (d, J = 8.1 Hz, 3H). ^{13}C NMR (126 MHz, $CDCl_3$) δ (ppm): 170.69, 166.30, 160.80, 147.07, 146.67, 143.38, 136.66, 136.07, 135.77, 129.25, 127.76, 126.13, 123.85, 121.93, 119.33, 118.84, 66.51, 55.72, 46.40. Anal. calcd for $C_{99}H_{78}IrN_3O_6$: C, 74.41; H, 4.92; N, 2.63. Found: C, 74.39; H, 4.92; N, 2.84.

NMR spectra of the synthesized compounds

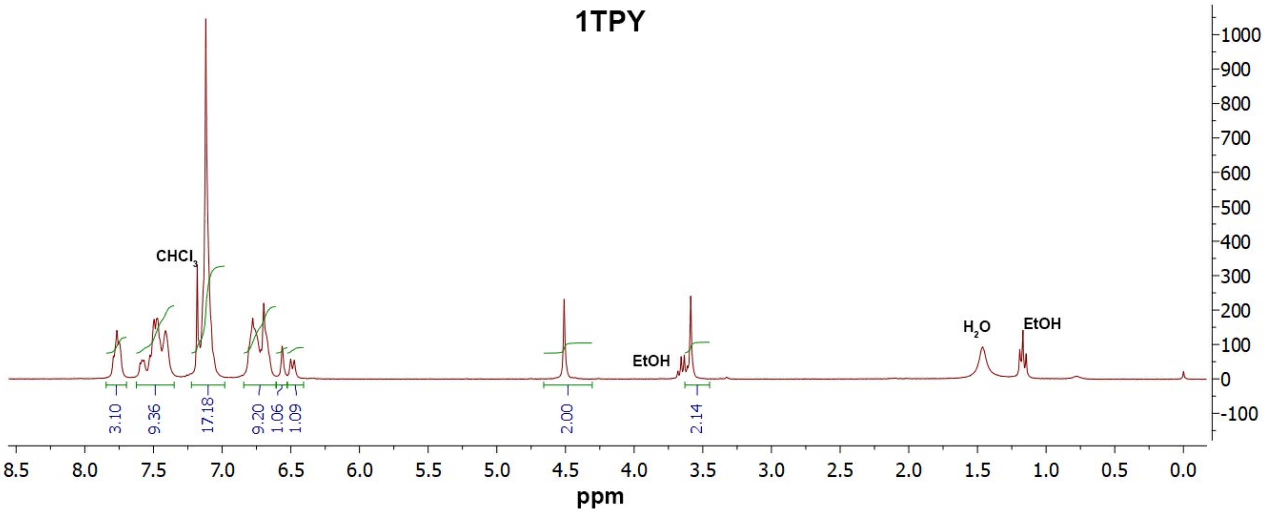

^{1}H NMR spectra of **1TPY** ($CDCl_3$).

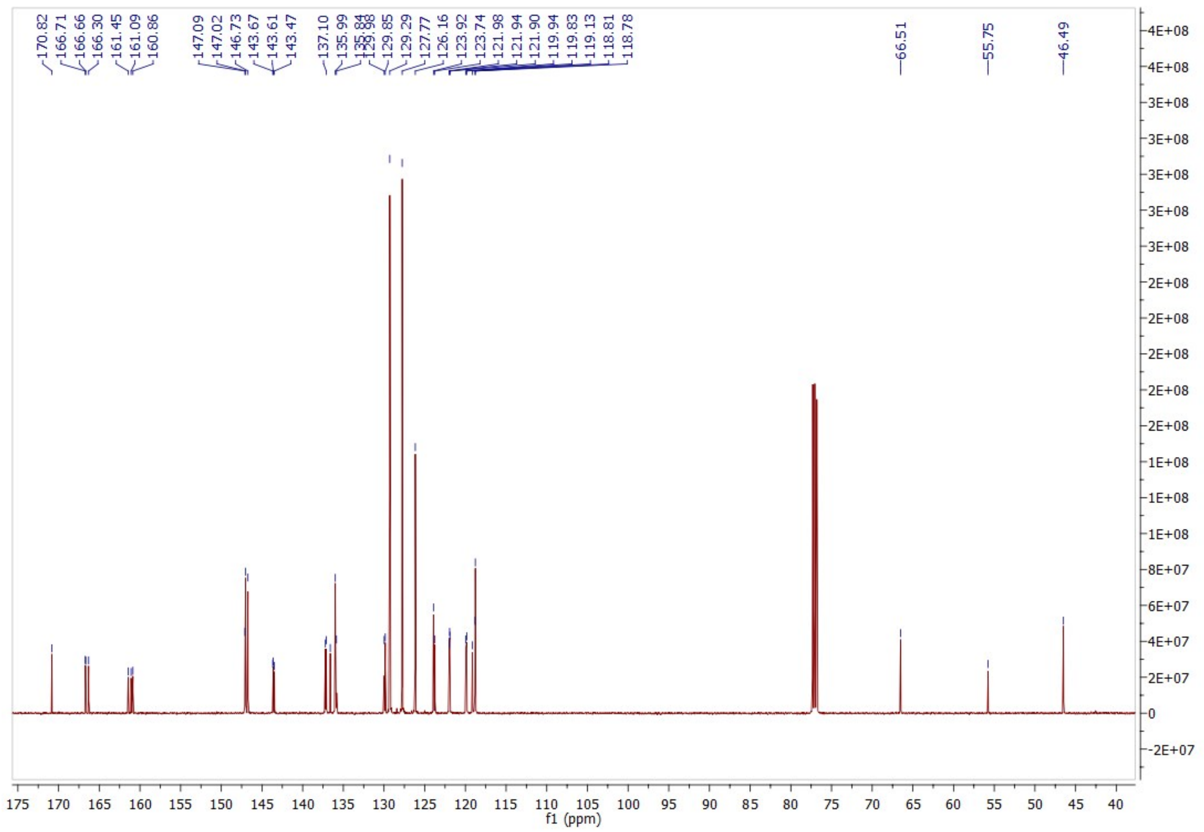

^{13}C spectra of **1TPY** ($CDCl_3$).

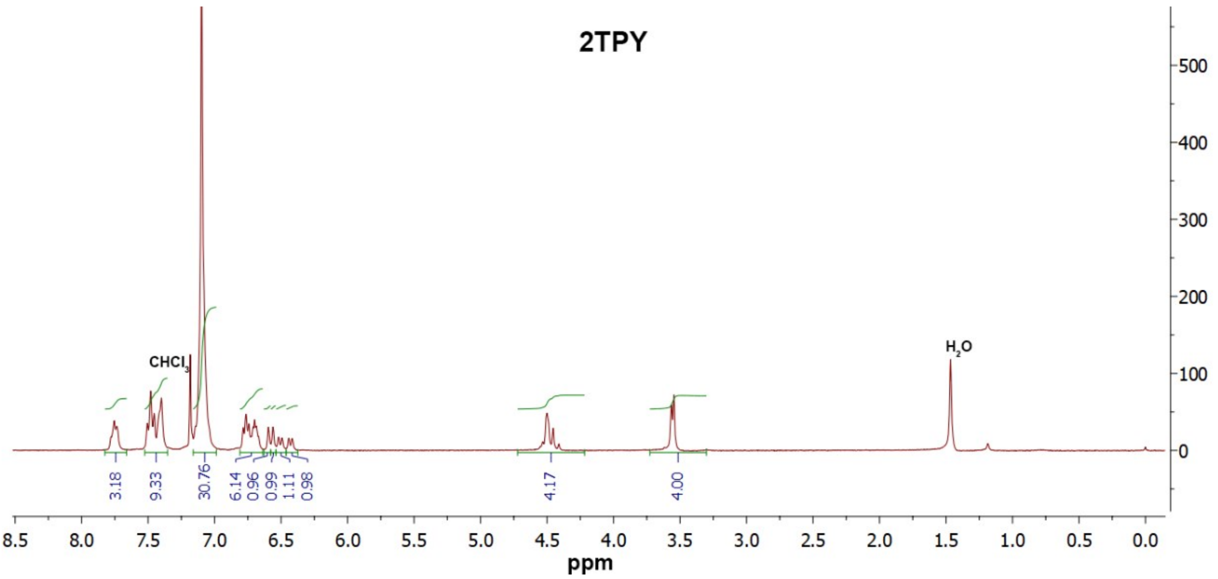

^{1}H NMR spectra of **2TPY** ($CDCl_3$).

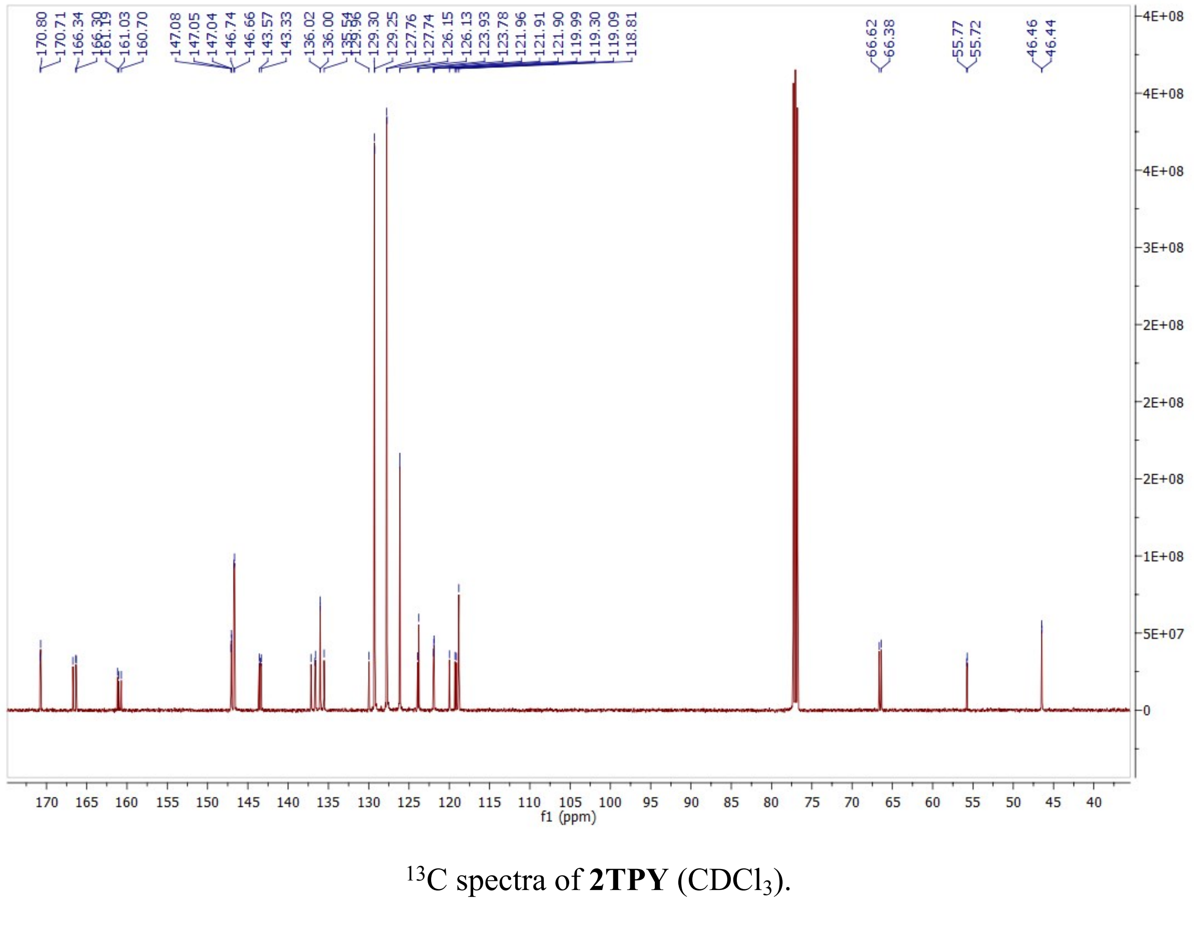

^{13}C spectra of **2TPY** ($CDCl_3$).

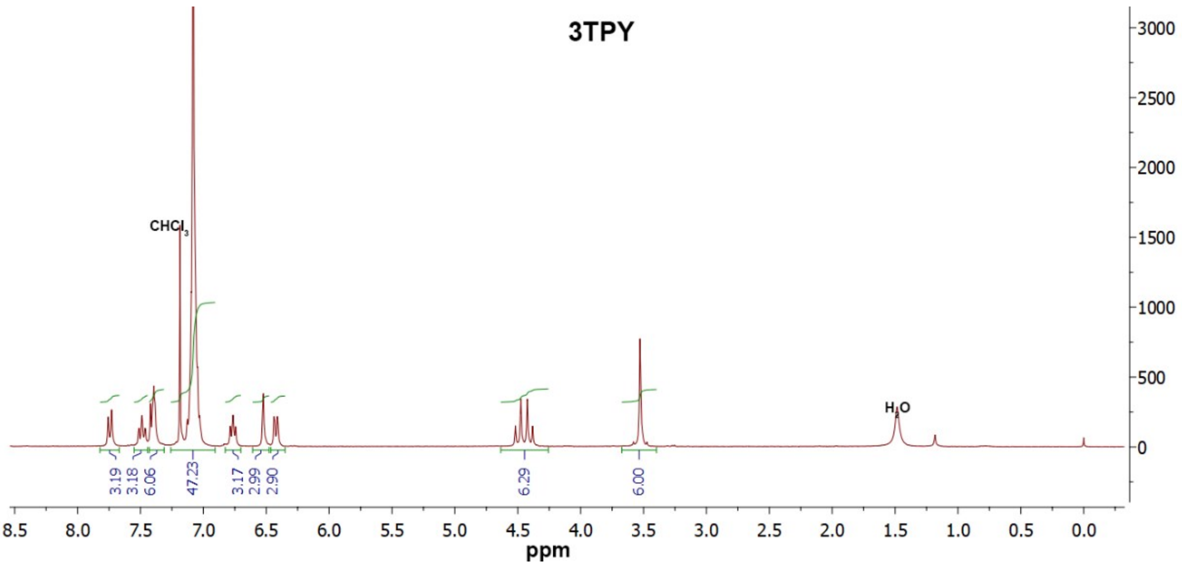

^{1}H NMR spectra of 3TPY ($CDCl_3$).

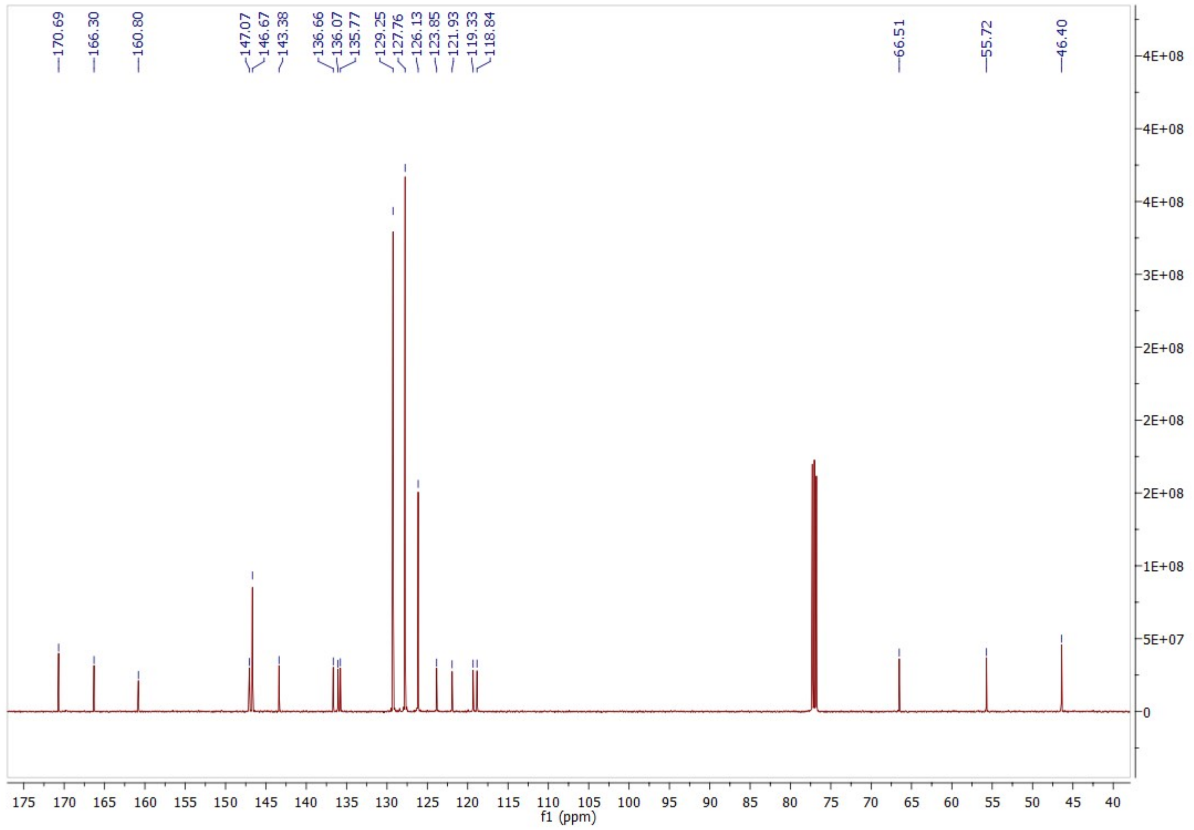

^{13}C spectra of **3TPY** ($CDCl_3$).

OLED preparation and characterization

Sandwich-type devices with the pixel size of 16mm^2 were prepared with the following structure: ITO/PEDOT:PSS(40 nm)/emitting layer(50 nm)/TPBi (20 nm)/LiF(1 nm)/Al(100 nm), where poly(3,4-ethylenedioxythiophene) poly(styrenesulfonate) (PEDOT:PSS), 2,2',2"-(1,3,5-benzinetriyl)-tris(1-phenyl-1-H-benzimidazole) (TPBi) and LiF were used as hole-injection, electron transport and electron-injection layers, respectively. Emitting layer consisted of iridium(III) complex ($Ir(ppy)_3$ core content 7 wt% in the all cases), mixed with poly(9-vinylcarbazole) (PVK; *Sigma Aldrich* 368350) or in a blend PVK: 1,3-bis[2-(4-tert-butylphenyl)-1,3,4-oxadiazo-5-yl]benzene (OXD-7; *Ossila M451*) with a mass ratio 70:30 wt%, or in a blend PVK:OXD-7 with a mass ratio 30:70 wt%. Indium tin oxide (ITO) glass (*Präzisions Glas & Optik GmbH*) with a sheet resistivity of 15 Ω/square was used as a substrate. A 12 mm wide ITO strip line was made by a wet etching. ITO substrates were cleaned by the following method: sonicated in $CHCl_3$; sonicated in acetone; 2x rinsed with deionized (DI) water; sonicated in water with 3 vol% of *Hellmanex II* detergent; rinsed with DI water; sonicated in DI water and isopropyl alcohol. Before the deposition of PEDOT:PSS, ITO glass was blown dry with nitrogen and treated with UV-ozone for 20 min. ITO layer was covered with PEDOT:PSS (*H.C. Starck*, Al4083) using spin-coater *Laurell WS650*. Rotation lasted for 1 min at speed 2000 rpm. The sample was moved in a glovebox and heated at 125 °C for 20 min. The solution of emitting layer compounds in tetrahydrofuran with a concentration of 5 mg/mL was spin-coated on the PEDOT:PSS layer at 2000 rpm for 40 s and heated at 100 °C for 10 min afterwards. Without the exposure to air, the samples were moved from glovebox to vacuum chamber for thermal evaporation of electron transport TPBi (*Sigma Aldrich* 806781) layer, electron-injection LiF (*Sigma Aldrich* 449903) layer and Al electrode (pressure $6 \cdot 10^{-6}$ Torr). The deposition speed was 1 Å/s, 0.1 Å/s and 5 Å/s for TPBi, LiF and Al, respectively. The current–voltage characteristics of the OLEDs were measured with *Keithley 2450*

SourceMeter. The electroluminescence brightness was measured with *Konica Minolta Luminance and Color Meter CS-150*. In the case of devices with PVK:OXD-7 (3:7) host system the voltage step of 0.2 volts was used for the measurements, while in the other cases the step was 0.5 V. In the former case the observed current density was unstable if the voltage step was 0.5 V, and the 0.2 V step was used to overcome this.

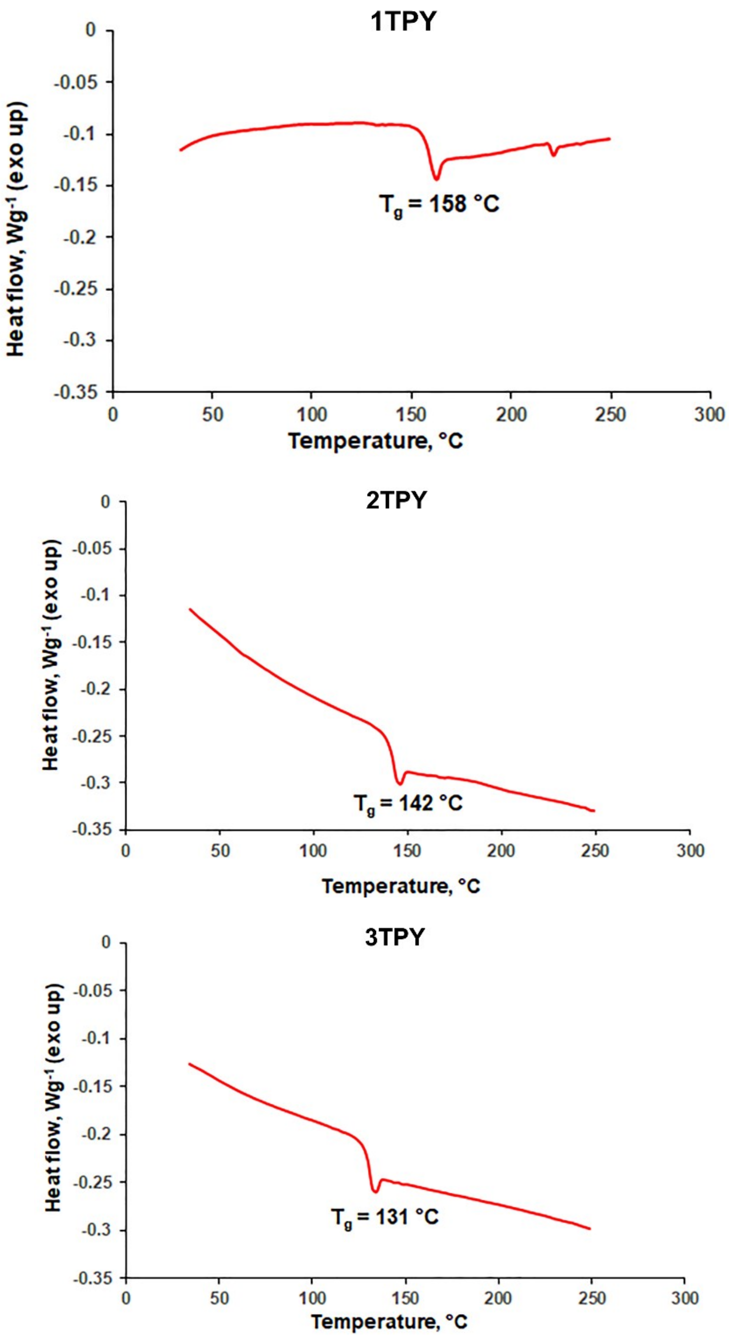

Figure S1. DSC scans of the materials with outlined T_g values.

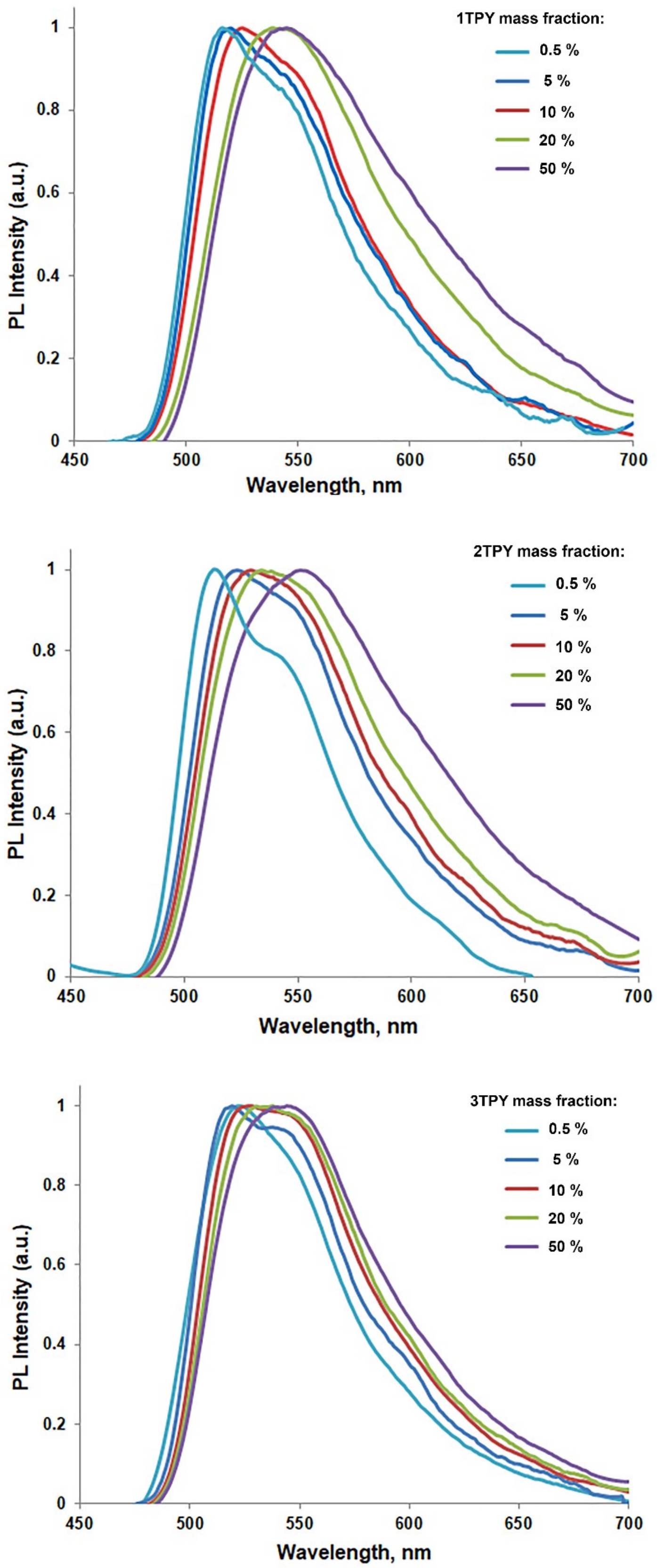

Figure S2. Film emission spectra of emitter-PVK guest-host systems with different emitter mass fractions.

Table S1. Emission band transformations in PVK host with increasing emitter concentration.

	**Emitter mass fraction (wt%)**				
	0.5	5	10	20	50
**Compound**	$\lambda_{em.\ max}$, nm (ΔFWHM,[a] cm^{-1})				
3TPY	514 (0)	522 (100)	528 (217)	538 (197)	544 (357)
2TPY	513 (0)	525 (227)	529 (295)	534 (493)	551 (857)
1TPY	514 (0)	525 (101)	520 (161)	539 (384)	545 (650)

[a] Relative broadening of the emission bands in comparison to 0.5 wt% sample.

References

(1) Lamansky, S.; Djurovich, P.; Murphy, D.; Abdel-Razzaq, F.; Kwong, R.; Tsyba, I.; Bortz, M.; Mui, B.; Bau, R.; Thompson, M. E. Synthesis and Characterization of Phosphorescent Cyclometalated Iridium Complexes. *Inorg. Chem.* **2001**, *40* (7), 1704–1711. https://doi.org/10.1021/ic0008969.

(2) Bettington, S.; Thompson, A. L.; Beeby, A.; Goeta, A. E. Di-μ-Chloro-Bis{bis[4-(2-Pyridyl)Benzaldehyde-κ 2 C 2 , N ′]Iridium} Dichloromethane Sesquisolvate. *Acta Crystallogr. Sect. E Struct. Reports Online* **2004**, *60* (6), m827–m829. https://doi.org/10.1107/S1600536804011663.
